# The Effects of Low-Carbohydrate Diets on Psychosocial Outcomes in Obesity/Overweight: A Systematic Review of Randomized, Controlled Studies

**DOI:** 10.3390/nu8070402

**Published:** 2016-06-29

**Authors:** Marwan El Ghoch, Simona Calugi, Riccardo Dalle Grave

**Affiliations:** Department of Eating and Weight Disorders, Villa Garda Hospital, Via Montebaldo, 89, Garda I-37016, Italy; si.calugi@gmail.com (S.C.); rdalleg@gmail.com (R.D.G.)

**Keywords:** obesity, overweight, weight loss, low-carbohydrate diets, psychosocial outcomes, mood, depression, anxiety

## Abstract

Background: Little is known about the relative psychosocial effects of carbohydrate reduction in comparison to other weight-loss diets in subjects receiving treatment for obesity/overweight. We, therefore, set out to conduct a systematic review of the psychosocial outcomes of such patients, treated by means of either a low-carbohydrate diet or an isocaloric diet of other macronutrient composition. Methods: Literature searches, study selection, method development, and quality appraisal were performed independently by two authors, and data were synthesized using a narrative approach, in accordance with the Preferred Reporting Items for Systematic Review and Meta-Analyses (PRISMA) guidelines. Results: Eight randomized controlled studies met the inclusion criteria, and their subsequent analysis revealed that improvements in psychological and social outcomes do occur during short- and long-term weight loss programmes, but that low-carbohydrate diets have no greater effect on psychosocial outcomes when compared to diets of different macronutrient composition at either short- or long-term follow-up (one-year). However, the lack of studies with longer duration follow-up, and the absence of data in the adolescent population limit the generalizability of our findings. Conclusion: The short- and long-term improvements in psychosocial outcomes seen in patients undergoing weight-loss treatment appear to be independent of the macronutrient composition of their diet.

## 1. Introduction

In the field of obesity treatment, the psychological effects of diet and weight loss have been a continuing source of debate and controversy. An early review of studies published before the 1970s found an association between dieting and negative emotional responses [1], whereas later studies have reported improvements or no change in the symptoms of depression and anxiety in patients with obesity managed by behavioural weight-loss treatment associated with either moderate [2,3,4,5] or severe caloric restriction [6,7,8], or the use of weight-loss drugs [9,10,11]. A lessening of psychological distress has also been found in a large observational study assessing the effect of dieting and moderate weight loss in treatment-seeking patients with obesity at several “real-world” medical obesity centres [12]. In addition, Look AHEAD, a large multicenter study comparing an intensive lifestyle intervention based on behaviour modification with a diabetes support and education (DSE) control intervention, has recently shown that the former is associated with a greater reduction in the incidence of depression symptoms and preservation of health-related quality of life than the latter [13].

The optimal macronutrient composition for weight loss in patients receiving treatment for obesity/overweight in the presence or absence of comorbidities is another subject requiring clarification [14], and few things have been the subject of such intense discussion as the efficiency of low-carbohydrate diets in comparison to diets of different macronutrient composition, particularly over the last two decades. Although several studies have shown the beneficial effects of low-carbohydrate diets on short- [15,16,17,18,19,20,21,22,23,24,25,26,27,28,29,30,31,32] and long-term [33,34] weight loss, body composition, and cardiovascular and metabolic features i.e., blood pressure, abdominal fat, triglyceridemia, High Density Lipoprotein (HDL) cholesterol, non-HDL cholesterol, fasting glucose, circulating insulin level, insulin sensitivity, HbA1c, and diabetes medication [17,18,19,20,21,22,23,25,29,31,34,35,36,37,38,39], their effect on psychosocial outcomes, in comparison to diets with other macronutrient compositions, remains unclear [40]. To our knowledge, the published literature on this topic has not yet undergone systematic review and we, therefore, set out to conduct one in accordance with the PICO process, as detailed below [41].

P-Population: subjects in the overweight or obesity categories, with or without comorbidities. I-Intervention: short- or long-term weight loss followed or not by a period of weight maintenance. C-Comparison: weight-loss programs involving low-carbohydrate diets as a treatment for obesity/overweight, as compared to any other diet. O-Outcome: changes in psychological and social profiles after weight loss.

## 2. Methods

Care was taken to adhere to the Preferred Reporting Items for Systematic Review and Meta-Analyses (PRISMA) guidelines in completion of this review [42]. PROSPERO Registry—the effects of “low-carbohydrate” diet on psychosocial outcomes in overweight and obese: a systematic review (CRD42016033703).

### 2.1. Inclusion and Exclusion Criteria

All studies evaluating changes in psychological and/or social parameters before and after weight loss achieved via low-carbohydrate diets were evaluated. Since randomized controlled studies provide strong evidence for the efficacy of healthcare interventions [43], we included studies that met the following criteria: (i) written in English; and (ii) described as randomized, a randomized trial (RCT), a randomized clinical trial, or an RCT study. No prospective or retrospective observational (analytical or descriptive), experimental or quasi-experimental, controlled or non-controlled studies, reviews, or cross-sectional, or non-original articles (i.e., case reports, editorials, letters to the editor, or book chapters) were included.

### 2.2. Information Source and Search Strategy

The literature search was performed independently and in duplicate by two authors. The PubMed database [44] was systematically screened using the following MeSH terms: #1 obesity; #2 overweight; #3 low-carbohydrate diets; #4 weight loss; #5 weight reduction; #6 weight maintenance; #7 psychosocial outcomes; #8 mood; #9 depression; and #10 anxiety. The following combinations were also applied as search parameters: (#1 OR #2) AND (#3) AND (#4 OR #5 OR #6) AND (#7 OR #8 OR #9 OR #10). Publication date was not considered an exclusion criterion for the purposes of this review.

### 2.3. Study Selection

Two authors independently screened the resulting articles for their methodology and appropriateness for inclusion. All included studies were assessed using risk-of-bias criteria [45,46]. Although ten criteria are generally used to assess the sufficiency of reporting, specifically the randomization method, allocation sequence concealment, participant blinding, outcome-assessor blinding, outcome measurement, interventionist training, withdrawals, intent-to-treat analyses, clustering, and baseline characteristics, we did not assess for participant blinding or outcome-assessor blinding criteria, since they are incompatible with the intervention in question. Studies were assigned a “Yes” for each applicable criterion they met, and a “No” for each they did not. Studies containing insufficient information to judge were indicated as “Not Reported”, and any disagreement was documented and resolved by discussion.

### 2.4. Data Collection Process and Data Items

Two authors independently assessed the title and abstract of each paper for language suitability and subject matter relevance, and then their quality of method and appropriateness for inclusion. Table 1 shows the first author, year of publication, sample gender, sample age, duration of follow-up, diet composition, psychosocial outcomes, and changes in outcomes of each study that passed these two rounds of screening.

### 2.5. Data Synthesis

This systematic review is presented as a narrative synthesis [47], since a meaningful meta-analysis could not be performed due to the lack of homogeneity among the resulting studies. In particular, studies varied in both the types of psychosocial outcomes were assessed (i.e., mood, depression, anxiety, etc.), and the tools used to assess them.

There is no agreement or set definition amongst clinicians and researchers regarding the carbohydrate thresholds for classifying low-carbohydrate diets. However, for the purposes of this review we adopted the system proposed by Accurso and colleagues [48].

Moderately-low-carbohydrate diet: carbohydrate makes up ~26%–45% of energy intake.

Low-carbohydrate diet: carbohydrate makes up less than 6%–26% of energy intake.

Very-low-carbohydrate diet: carbohydrate makes up less than 6% of energy intake.

## 3. Results

The initial search retrieved 7227 papers. After the first round of screening, 2840 papers were excluded for being in a language other than English, not being conducted on humans, and/or unavailability of either abstract or full text. The second round of screening excluded 1016 articles based on the type of the paper; editorials, case reports, and review articles, including narrative, systematic, and meta-analysis reviews were excluded. The third round of screening excluded 3457 articles for having no bearing on overweight or obesity, or dealing with participants in the overweight and obesity categories, but failing to consider psychosocial outcomes, diet composition, or comparisons between diets of different compositions. At this point, 59 articles of the remaining 70 papers were excluded because, although comparisons between diet compositions were available, there was no evaluation of changes in any psychological or social outcomes.

**Figure 1:**
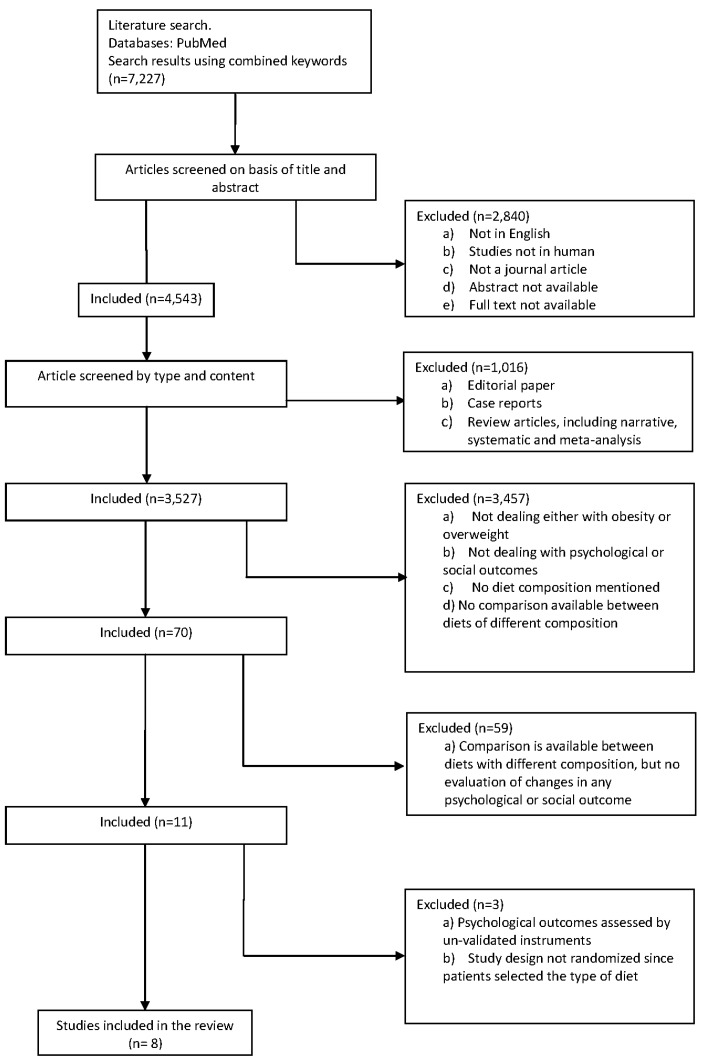
Flowchart summarizing the study selection procedure.

Finally, of the 11 remaining papers, three were eliminated due to a methodological limitations, either (i) psychological outcomes assessed by unvalidated instruments [26,49]; or (ii) randomization compromised by the patients selecting the type of diet to undergo [50] (Figure 1). Thus, at the end of the screening process, only eight articles were available for systematic review and narrative analysis.

### 3.1. The Short-Term Psychosocial Effects of a Low-Carbohydrate Diet

In 1982, Rosen et al. [51] were the first to compare the effects on mood of six weeks of carbohydrate restriction (827 kcal; 35% protein, 64% fat, 1% carbohydrate) with respect to a carbohydrate-containing diet (827 kcal; 35% protein, 36% fat, 29% carbohydrate). In that small study, changes in psychological symptomatology were assessed in eight females with obesity (mean BMI: 31 kg/m^2^), and an age ranging between 25 and 33 years. State Anxiety Inventory (STAI-T) [58] and Beck Depression Inventory (BDI) [59] scores at baseline and at the end of the experiment showed no mood elevation associated with the carbohydrate-restricted diet, and no difference in psychological effects with respect to the isocaloric carbohydrate-containing diet.

In 1985, Rosen et al. [52] assessed changes in several psychological variables in 20 patients (19 females and one male) between 20 and 38 years (mean age: 29.2 ± 5.12 years) who spent eight weeks alternating between two-week periods of a minimal-carbohydrate diet (800 kcal; 58% protein and 42% fat by weight) and a carbohydrate-supplemented diet (1000 kcal; 42% protein, 30% fat, and 28% carbohydrate) as a treatment for obesity/overweight. Changes in anxiety, depression, and hostility associated with the two diets were assessed using the three respective scales of the Multiple Affect Adjective Checklist (MAACL) [60], and psychological wellbeing and self-esteem with the Tennessee Self Concept Scale (TSC) [61]. Participants on both diets lost significant weight (~11 kg) across the eight weeks of dieting, and both groups showed reduced appetite and elevated psychological wellbeing in the initial two weeks. Afterwards, however, appetite and mood returned to baseline levels, and no association was found between subsequent changes in psychological reactions to dieting and the type of diet.

In 2007, Halyburton et al. [27] compared the effects on psychological wellbeing of a low-carbohydrate, high-fat, energy-restricted diet (approximately 6–7 megajoule, 30% deficit) with an isocaloric conventional high-carbohydrate, low-fat diet, both administered over eight weeks as treatments for obesity/overweight in 93 men and women (BMI range: 26–43 kg/m^2^, age range between 24 and 64 years). Psychological wellbeing was measured at baseline and fortnightly using the BDI [59], Spielberger State Inventory (SAI) [62], Profile of Mood States (POMS), [63] and Total Mood Disturbance Scores (TMDS). In this sample, although both groups showed improvements in psychological wellbeing, with the greatest effect occurring during the first two weeks, there was no significant difference between groups at the end of the study.

In 2014, Saslow et al. [54] assessed the effect on psychological outcomes of a three-month very-low-carbohydrate, high-fat diet (*n* = 16) against that of a moderate-carbohydrate, low-fat diet (*n* = 18) in 34 males and females diagnosed with obesity/overweight and type-2 diabetes or pre-diabetes. Psychological outcomes were assessed on the Diabetes Distress Scale, which measures the emotional upset related to having diabetes; the 20-item Centre for Epidemiologic Studies Depression Scale (CES-D) [64], which assesses aspects of depressive mood; the Dutch Eating Behaviour Scale [65], which describes eating in response to emotions (such as anger or irritation); the Body Responsiveness Questionnaire [66], which measures the importance of interoceptive awareness, and perceived disconnection between psychological and physical states; and the Three-Factor Eating Questionnaire, which assesses hunger, cognitive restraint, and disinhibited eating [67]. Despite the non-significant difference in weight loss between the two groups of dieters across the three months, the very-low-carbohydrate, high-fat diet improved glycemic control in type-2 diabetes, allowing the doses of diabetes medications to be reduced. However, no significant differences in psychological outcomes were observed between the two diets.

In 2007 Galletly et al. [53] assessed the effects of 16 weeks of high-protein, low-carbohydrate diet or low-protein, high-carbohydrate diet, randomly assigned to 28 women with obesity and polycystic ovary syndrome, matched for age, weight, and whether or not they were trying to conceive. The Hospital Anxiety and Depression (HAD) Scale [68] and the Rosenberg Self-Esteem Scale (RSES) [69] were administered at the beginning and end of the diet and, though there was no difference in weight loss between the groups, participants allocated to the high-protein, low-carbohydrate diet displayed a significantly greater improvement in depression and self-esteem with respect to the low-protein, high-carbohydrate diet group. However, the authors suggested that this discrepancy with the findings reported above could be related to the effect of the high protein content of the diet rather than the low carbohydrate content. 

### 3.2. The Long-Term Psychosocial Effects of a Low-Carbohydrate Diet

In 2009, Brinkworth et al. [55] assessed 106 participants randomly assigned to a very-low-carbohydrate, high-fat diet (*n* = 57) or an isocaloric high-carbohydrate, low-fat diet (*n* = 61) for one year as a treatment for obesity/overweight (mean BMI of 33.7 ± 0.4 kg/m^2^). The psychological wellbeing of the participants, aged between 24 and 64 years (mean age 50.0 ± 0.8 years), was assessed using the POMS [63], BDI [59], and SAI [62] at weeks zero, eight, 24, and 52. Although no significant difference in weight loss was observed between the two groups after one year, participants allocated to the high-carbohydrate, low-fat diet showed greater improvements in anxiety, depression, total mood disturbance, anger-hostility, confusion-bewilderment, and depression-dejection mood states with respect to those on the very-low-carbohydrate, high-fat diet.

In 2013, Dalle Grave et al. [56] compared the long-term effects of a high-carbohydrate low-protein diet and low-carbohydrate, high-protein diet in patients undergoing cognitive behavioural therapy (CBT). A total of 88 individuals with class II and III obesity were randomized to the two types of diet over one year. Psychosocial outcomes were assessed at baseline, after three weeks of inpatient treatment, and at 27 weeks and one year of follow-up by means of the Body Uneasiness Test-A (BUT) [70], Binge Eating Scale (BES) [71], BDI [59], and Beck Anxiety Inventory (BAI) [72]. All psychosocial measures improved significantly from baseline to one year, with no differences between the two diet groups.

In 2016, Brinkworth et al. [57] examined the long-term effects of a very-low-carbohydrate diet on psychological health in 115 adults with obesity and type 2 diabetes aged between 35 to 68 years (mean age: 58.5 ± 7.1 years). Participants were randomized to consume either a low-carbohydrate energy-restricted diet (*n* = 58) or an isocaloric high-carbohydrate (*n* = 57) equivalent, both combined with a supervised exercise program, for one year. Psychological mood states and wellbeing were assessed by means of the POMS [63], BDI [59], and SAI [62]; the diabetes-specific emotional distress via the Problem Areas in Diabetes (PAID) Questionnaire [73]; and the quality of life with the QoLDiabetes-39 (D-39) [74]. Significant improvements over time in BDI, POMS, PAID, and D-39 scores were seen in both groups, but the macronutrient composition had no effect on the response in terms of the psychological outcomes assessed. The authors, therefore, concluded that either a low- or a high-carbohydrate diet, within a lifestyle modification program that includes exercise training, improves psychological wellbeing to a similar extent, confirming the finding reported by Dalle Grave et al. [56].

## 4. Discussion

The aim of this review was to provide benchmark data on the effects of low-carbohydrate diets, as compared to diets of other macronutrient composition, on psychosocial outcomes in people with excess weight/obesity. The review, which included eight randomized, controlled studies judged objectively to be of high quality (Table 2), yielded three main findings.

The first finding was that short- [27,52] and long-term [56,57] weight-loss treatments based on dietary restriction are associated with significant improvements in psychological and social outcomes. The second finding is that low-carbohydrate diets are associated with similar improvements in psychological outcomes at short-term follow-up (i.e., from three weeks to four months) to other types of diet of different macronutrient composition [27,51,52,54]. Only one short-term study [53] reported different findings, but in that case it was not clear whether the significantly greater improvements in depression and self-esteem found to be associated with the high-protein low-carbohydrate diet were related to the low carbohydrate content or the higher protein content in this diet. The third finding of the review is that long-term studies (i.e., one year follow-up) [55,56,57] showed no additional benefits of low-carbohydrate diets in terms of psychosocial outcomes when compared with diets of other macronutrient composition [55,56,57].

However, there are two major concerns regarding the above findings. The first is that no study was set up to specifically assess the separate effects on psychosocial outcomes of weight loss and carbohydrate restriction. Nevertheless, the included studies that did report both the psychological outcome and weight loss, [52,53,54,55,56,57] found no significant differences in weight-loss rate between the types of diet employed (low-carbohydrate diets vs. other diets of different composition). As this would tend to indirectly exclude the influence of weight loss on psychological outcomes, the resulting data do permit the effects of diet composition to be assessed, albeit with some caution. Furthermore, despite the wide heterogeneity in the energy-restricted diets featuring other macronutrient compositions (i.e., in some studies carbohydrates were replaced prevalently by proteins [52,56], whereas in others by fats [27,51,55,57]), no distinct differences between these and the low-carbohydrate diets were noted in terms of psychosocial outcomes. Despite these legitimate concerns, therefore, the three main findings of our review may be considered robust, as they derive exclusively from well-designed studies (randomized, controlled studies).

Indeed, this review has several strengths. Firstly, we strictly adhered to the Preferred Reporting Items for Systematic Review and Meta-Analyses (PRISMA) guidelines, and this methodological robustness lends weight to the validity of the conclusions; secondly, the studies included in this review were extremely well designed, featuring both randomized samples and appropriate control groups; thirdly, the instruments used in all studies to assess the psychosocial outcomes have been amply validated and acknowledged in both clinical and research settings.

That being said, the review does have some limitations. In particular we were prevented from performing a meta-analysis due to the lack of homogeneity between psychosocial-outcome assessment and scoring. Furthermore, both a lack of studies in adolescents, and the unavailability of studies that exceeded one year of follow-up in adults limit the generalizability of our findings to the entire obese/overweight population. Finally, as we were unable to analyse the quality of carbohydrates in the low-carbohydrate diets administered to the participants, we can offer no conclusions as to the potential influence of this factor on psychosocial outcomes.

## 5. Conclusions

Although the beneficial effect of low-carbohydrate diets on weight loss and weight maintenance in the short- [15,16,17,18,19,20,21,22,23,24,25,26,27,28,29,30,31,32] and intermediate-long terms [33,34] are well documented, the psychosocial improvements associated with weight loss seem not to be influenced by the macronutrient composition of the diet. However, to enable these findings to be generalized to the entire overweight/obesity population, future studies should be performed with longer follow-up periods in both adults and adolescents.

## Figures and Tables

**Table 1:** Studies included in the systematic review.

First Author	Year	Gender	Duration	Sample	Age Range	Diet Composition	Outcome Measure	Change in Outcome
**Short-Term Studies**								
Rosen et al. [51]	1982	F	8 weeks	Eight females treated for obesity	25–33 years	**CHO**: 35% proteins, 36% fats, 29% carbs **NO CHO**: 35% proteins, 64% fats, 1% carbs	STAI-T BDI	No difference in psychological effects between the two diets
Rosen et al. [52]	1985	F and M	8 weeks	19 females and 1 male treated for obesity/overweight	20–38 years	**MCD**: 58% proteins, 42% fats, 0% carbs **CCD**: 42% proteins, 30% fats, 28% carbs	MAACL TSC	No difference in psychological effects between the two diets
Halyburton et al. [27]	2007	F and M	8 weeks	121 men and women treated for obesity/overweight	24–64 years	**HFLC**: 35% proteins, 61% fats, 4% carbs **LFHC**: 24% proteins, 30% fats, 46% carbs	SAI POMS TMDS	Similar effects of diets on psychological well-being effects
Galletly et al. [53]	2007	F	4 months	*n* = 14 LPHC *n* = 14 HPLC	33.0 ± 1.2 years 32.0 ± 1.2 years	**LPHC**: 15% proteins, 30% fats, 55% carbs **HPLC**: 30% proteins, 30% fats, 40% carbs	HAD RSES	Significant improvement in depression and self-esteem in the In the HPLC group
Saslow et al. [54]	2014	F and M	3 months	34 patients with type-2 diabetes mellitus or pre-diabetes treated for obesity/overweight	64.8 ± 7.7 years 55.1 ± 13.5 years	**LCK**: 20–50 grams of carbohydrate day **MCCR**: 45%–50% carbohydrate	Diabetes Distress Scale CES-D Dutch Eating Behaviour Scale Body Responsiveness-Q Three-Factor Eating-Q	No difference in psychological measures between the two diets
**Long Term Studies**								
Brinkworth et al. [55]	2009	F and M	1 year	Participants treated for obesity/overweight, *n* = 57 HFLC *n* = 61 LFHC	24–64 years	**HFLC**: 35% proteins, 61% fats, 4% carbs **LFHC**: 24% proteins, 30% fats, 46% carbs	STAI BDI POMS	Greater improvements in psychological outcomes for the LFHC diet compared with the LC diet
Dalle Grave et al. [56]	2013	F and M	1 year	88 class II–III obesity patients randomized to LPHC and HPLC	18–65 years	**LPHC**: 17% proteins, 20% fats, 63% carbs **HPLC**: 34% proteins, 20% fats, 46% carbs	BAI BDI BES BUT-GSI	All psychosocial measures decreased from baseline to 1 year, with no differences between groups
Brinkworth et al. [57]	2016	F and M	1 year	*n* = 58 LC *n* = 57 HC	35–68 years	**LC**: 28% proteins, 58% fat, 14% carbs **HC**: 17% proteins, 15% fat, 53% carbs	POMS BDI SAI PAID D-39	Improvements in psychosocial measures (POMS, BDI and PAID) after 1 year, with no differences between groups

Abbreviations. F: females; M: males; LC: low-carbohydrate; HC: high-carbohydrate; LPHC: low-protein, high-carbohydrate; HPLC: high-protein, low-carbohydrate; HFLC: high-fat, low-carbohydrate; LFHC: low-fat, high-carbohydrate; CHO: carbohydrate; MCD: minimal-carbohydrate diet; CCD: carbohydrate-containing diet; LCK: low-carbohydrate ketogenic; MCCR: medium-carbohydrate, low fat, calorie-restricted, carbohydrate-counting diet; STAI-T: State Anxiety inventory; BDI: Beck Depression Inventory; MAACL: Multiple Affect Adjective Checklist; TSC: Tennessee Self Concept Scale; SAI: Spielberger State Inventory; POMS: Profile Of Mood States; TMDS: Total Mood Disturbance Scores; HAD: Hospital Anxiety and Depression; RSES: Rosenberg Self Esteem; BAI: Beck Anxiety Inventory; BES: Binge Eating Scale; BUT: Body Uneasiness Test; PAID: Problem Areas in Diabetes; D-39: QoLDiabetes-39.

**Table 2:** Risk-of-bias criteria.

Authors	Rosen 1982 [51]	Rosen 1985 [52]	Halyburton 2007 [27]	Galletly 2007 [53]	Brinkworth 2009 [55]	Dalle Grave 2013 [56]	Saslow 2014 [54]	Brinkworth 2016 [57]
Was the method of randomization to groups appropriate?	+	+	+	+	+	+	+	+
Was the allocation sequence concealed from those assigning patients to groups?	nr	nr	nr	nr	nr	nr	+	+
Was the outcome measurement performed in the same manner with similar intensity in all groups being compared?	+	+	+	+	+	+	+	+
Were similarly trained individuals administering the intervention across groups?	+	+	+	+	+	+	+	+
Were all the withdrawals described?	−	−	+	−	+	+	−	+
Were all originally randomized participants analysed in the groups they were assigned to (i.e., an intention-to-treat analysis)?	−	−	+	−	+	+	+	+
Was clustering at the group level accounted for in the analyses?	−	−	nr	nr	nr	nr	−	+
Were the groups similar at baseline?	nr	+	+	+	+	+	+	+

Yes: +; No: −; Not reported: nr.
